# Mechanistic Insights into Lysine Cyclodeaminase Catalysis

**DOI:** 10.1021/acsomega.6c01676

**Published:** 2026-05-22

**Authors:** Yao Wei, Beatrice Rassati, Uliano Guerrini, Francesca Paradisi, Ivano Eberini

**Affiliations:** † Dipartimento di Scienze Farmacologiche e Biomolecolari “Rodolfo Paoletti”, 9304 Università degli Studi di Milano, Via Giuseppe Balzaretti 9, 20133 Milano, Italy; ‡ Department of Chemistry, Biochemistry and Pharmaceutical Sciences, 27210 University of Bern, Freiestrasse 3, 3012 Bern, Switzerland

## Abstract

Lysine cyclodeaminase (LCD) catalyzes the conversion of l-lysine into l-pipecolic acid, a key building block for food additives and pharmaceutical intermediates. Despite its industrial relevance, LCD displays a narrow substrate scope, efficiently converting l-lysine, while bulkier derivatives such as l-lysine ethyl ester fail to undergo productive biotransformation. To elucidate the molecular origin of this selectivity and define the catalytic mechanism, we combined molecular docking, substrate tunnel engineering, classical molecular dynamics, well-tempered metadynamics simulations, and experimental validation. Computational analyses show that both l-lysine and l-lysine ethyl ester can access and bind within the LCD active site, and tunnel engineering produced LCD variants (I61V–I94V–D236C and I61V–I94V–E264T) with improved tunnel properties. However, experimental assays demonstrated that these variants did not acquire catalytic activity toward l-lysine ethyl ester. Mechanistic simulations reveal that the proposed l-lysine iminium intermediate consistently adopts low-energy, cyclization-competent conformations in which the nucleophilic Nε and reactive Cα atoms achieve near-attack geometries. In contrast, the proposed l-lysine ethyl ester iminium intermediate populates higher-energy states with misaligned geometries and kinetically trapped conformations, suggesting difficult cyclization despite successful binding. These findings suggest that steric and dynamic constraints—rather than substrate access—could impact the catalysis of esterified substrates. This work establishes a mechanistic framework linking enzyme dynamics, substrate recognition, and catalytic efficiency, providing a foundation for rational LCD engineering aimed at expanding substrate scope and guiding future industrial applications.

## Graphical abstract

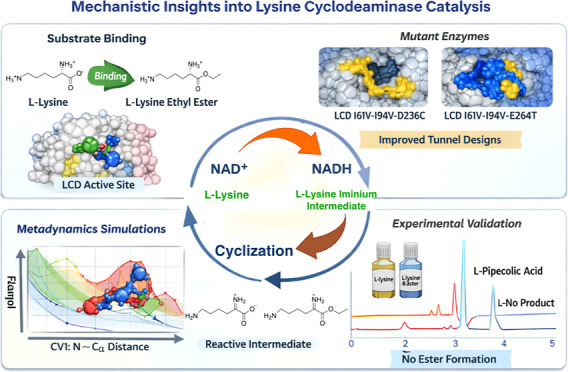

## 1 Introduction

l-Lysine cyclodeaminase (*Sp*LCD or LCD) from *Streptomyces pristinaespiralis* is a key enzyme with wide applications in the food and pharmaceutical industries due to its ability to catalyze the conversion of l-lysine into l-pipecolic acid. This reaction is important for producing food additives that influence flavor, color, and nutritional content, as well as pharmaceutical intermediates and amino-acid derivatives used in drug synthesis, making LCD a valuable biocatalyst in industrial biotechnology.^1,2^ l-Pipecolic acid is a nonproteinogenic amino acid widely used as a building block for the synthesis of pharmaceutical compounds, including the immunosuppressor rapamycin and the local anesthetic mepivacaine.^3–5^ Traditional chemical syntheses of l-pipecolic acid require multiple steps and harsh conditions, often resulting in limited yields and poor enantiomeric excess.^6^

Despite its biotechnological relevance, LCD exhibits a narrow substrate scope, efficiently catalyzing reactions only for molecules no larger than l-lysine.^7^ This limitation suggests that both the geometry of the active site and the dimensions of the substrate access tunnel may restrict effective substrate binding and subsequent catalytic conversion. From a mechanistic perspective, larger substrates such as l-lysine ethyl ester cannot be processed because of the presence of the ethyl group, which prevents LCD from completing the cyclodeamination reaction. Therefore, elucidating the structural and dynamic determinants that distinguish catalytically competent from incompetent substrate states is critical for guiding rational enzyme engineering aimed at expanding the substrate scope of LCD.

Computational approaches, including molecular docking and molecular dynamics simulations, can be used to assess whether the enzyme’s active site can appropriately accommodate different chemicals. Geometry-based tunnel analyses allow for the exploration of substrate transport pathways leading to the active site. The biotransformation of both l-lysine and l-lysine ethyl ester by designed LCD mutants has been validated through wet-lab experiments. Preliminary computational and experimental results indicate that neither the active site nor the substrate access tunnel represents the primary barrier to catalyzing the l-lysine ethyl ester. Subsequently, well-tempered metadynamics simulations enable efficient sampling of rare conformational transitions relevant to substrate binding and catalytic processes.^8^ Together, these methods offer a powerful framework to elucidate enzyme–substrate interactions at the atomic level.

By integrating molecular docking, tunnel analysis, metadynamics simulation, and experimental validation, this study uncovers the molecular mechanisms underlying substrate recognition and catalysis, offering insights that can guide the future engineering of LCD to enhance its catalytic performance and broaden its applicability in the food and pharmaceutical industries.

## 2 Results and Discussion

### 2.1 Molecular Docking to Construct LCD Protein–Ligand Complexes and Analyze Active-Site Binding

Since the available experimentally determined three-dimensional (3D) structures of LCD do not include the substrate l-lysine bound to its active site (PDB ID: 5GZL),^9^ the LCD protein–ligand complexes were generated using molecular docking. Combining the catalytic pathway of ornithine cyclodeaminase (OCD), a homologue of LCD, and the previously proposed catalytic pathway for LCD,^1^ the reaction can be supposed to proceed through an oxidative deamination by a direct cyclization mechanism (Figure 1).^10^ We further propose that the l-lysine ethyl ester can also be converted into the corresponding intermediate. The corresponding reaction schemes are shown in Figure 2. Accordingly, the iminium intermediates of both l-lysine and l-lysine ethyl ester were modeled by manually adjusting their structures. In addition, because the wet-lab experiments employed an LCD double mutant (I61V–I94V) that exhibited enhanced catalytic efficiency in converting l-lysine to l-pipecolic acid,^11,12^ the same mutation was introduced in silico to ensure consistency between computational and experimental systems.

**Figure 1:**
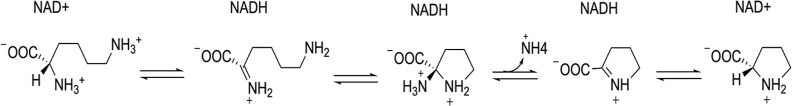
Proposed catalytic reaction pathway of LCD from substrate l-lysine to yield l-pipecolic acid.

**Figure 2:**
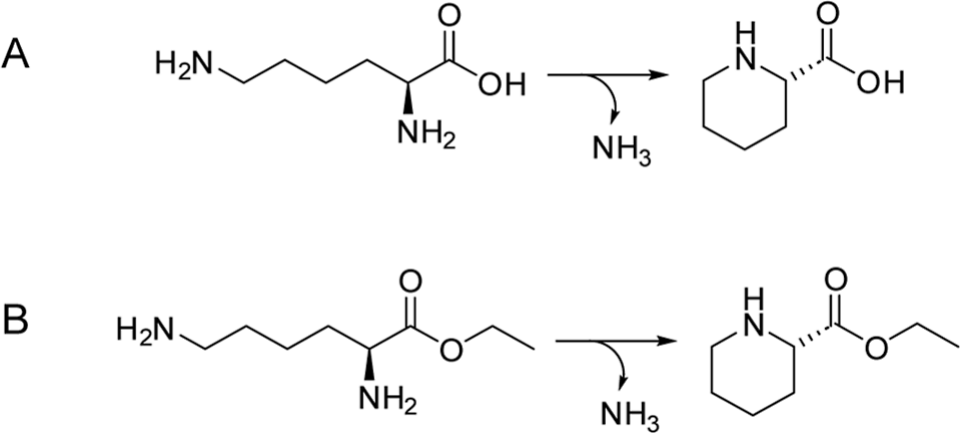
Simple reaction schemes of the target reactions to be catalyzed by *Sp*LCD variants to form l-pipecolic acid (A) or its ethyl ester derivative (B).

To investigate if the active site of LCD can structurally accommodate the bulkier l-lysine ethyl ester, guiding by the ligand-binding position observed in the crystallized LCD–l-pipecolic acid complex (PDB ID: 5GZM),^11^ induced-fit docking simulations were performed to obtain the LCD–l-lysine and LCD–l-lysine ethyl ester complexes.

The corresponding iminium intermediates were generated by manually modifying the l-lysine and l-lysine ethyl ester structures in the LCD active site. All these LCD protein—ligand complexes are shown in Figure 4. The LCD protein—ligand construction results further showed that both l-lysine ethyl ester and its iminium intermediate could be successfully accommodated in the active site, adopting positions comparable to those observed for l-lysine. These findings indicate that the LCD active site possesses sufficient steric capacity to bind larger substrates.

**Figure 3:**
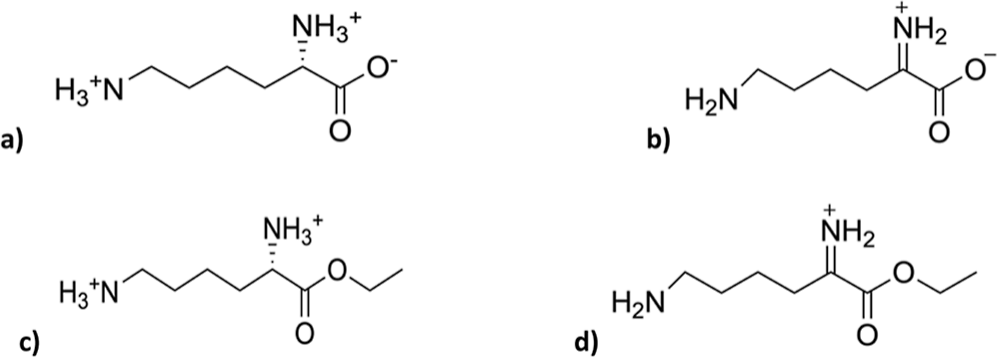
Chemical structures of (a) l-lysine; (b) l-lysine iminium intermediate; (c) l-lysine ethyl ester; and (d) l-lysine ethyl ester iminium intermediate.

**Figure 4:**
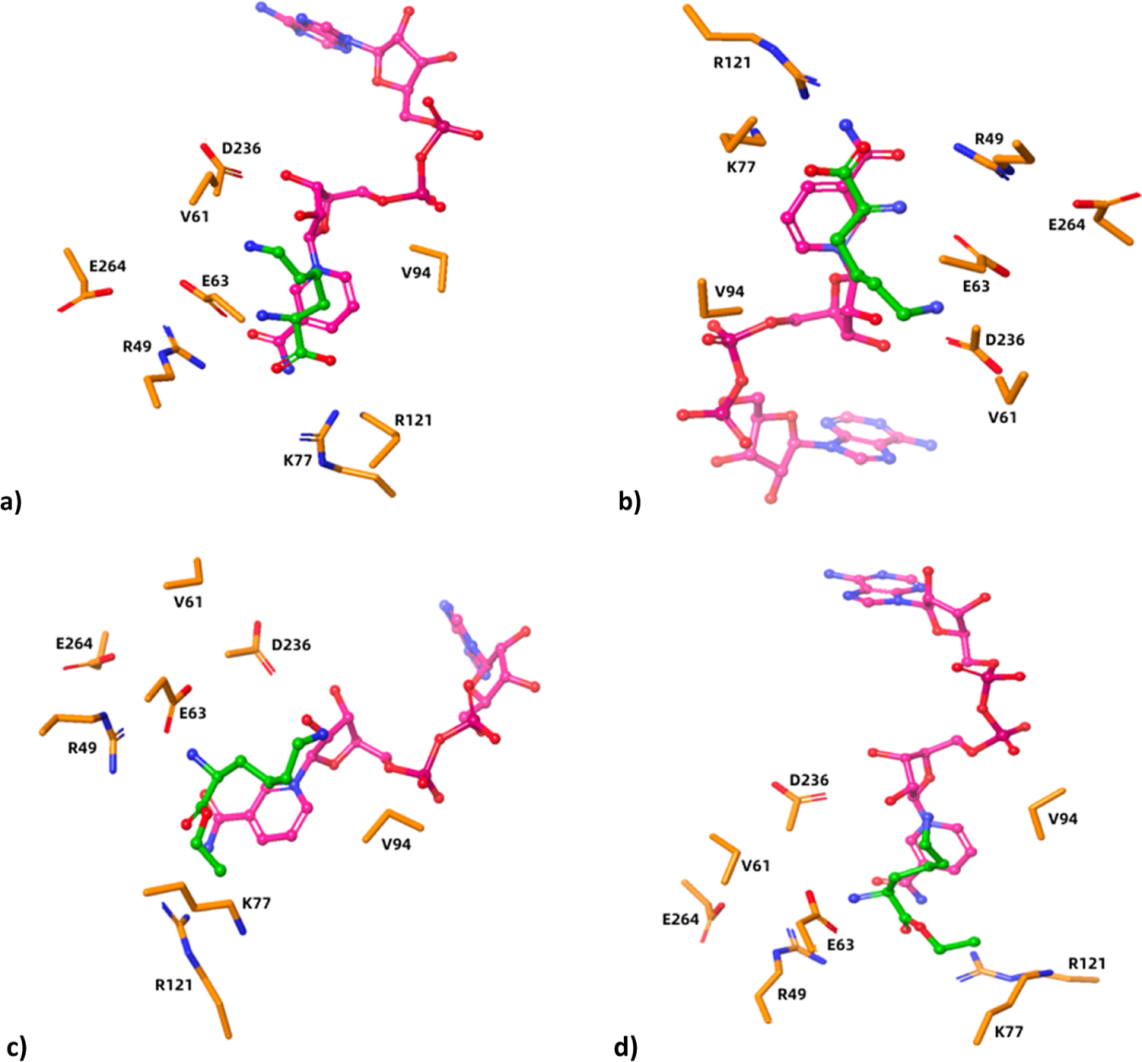
(a) LCD (NAD+)—l-lysine binding mode; (b) LCD (NADH)—l-lysine iminium intermediate; (c) LCD (NAD+)—l-lysine ethyl ester binding mode; (d) LCD (NADH)—l-lysine ethyl ester iminium intermediate. Ligands are shown in green, and NAD^+^ and NADH are shown in magenta.

We then conducted three replicate classical MD simulations for the four protein–ligand complexes. After equilibration, both protein Cα RMSD and ligand RMSD fluctuated within 1.0 Å, and the RMSF profiles of the four systems could be superimposed well during the simulations. These results indicate that all LCD protein–ligand complexes remained stable throughout the MD simulations. The corresponding protein Cα RMSD, ligand RMSD, and protein RMSF plots, obtained from the MD simulations, can be found in Figure S2.

To further evaluate the stability of the different LCD protein–ligand complexes, we performed MM/GBSA calculations on the ligands in their native and intermediate forms. The results are summarized in Table 1. The binding free energies indicate that all complexes are energetically favorable, with the l-lysine ethyl ester intermediate exhibiting the strongest binding (Δ*G* = −49.9 ± 1.5 kcal/mol), followed by l-lysine ethyl ester, l-lysine, and l-lysine intermediate. Van der Waals interactions were consistently favorable across all complexes and were strongest for the l-lysine ethyl ester intermediate, reflecting optimal shape complementarity and tight packing within the binding pocket. Hydrophobic interactions also increased along the series, indicating enhanced burial of nonpolar surfaces that further stabilized the ligands. Hydrogen bonding contributed modestly (−1.9 to −3.3 kcal/mol), playing a supportive rather than dominant role. Electrostatic interactions showed more complex behavior. In the LCD–l-lysine ethyl ester complex, highly favorable electrostatics (−140.2 kcal/mol) were nearly offset by an equally large desolvation penalty (+140.1 kcal/mol), resulting in minimal net contribution to binding. In contrast, the l-lysine ethyl ester intermediate exhibited more balanced electrostatics (−38.6 kcal/mol) with a smaller solvation penalty (+30.4 kcal/mol), contributing meaningfully to stabilization. The l-lysine intermediate showed unfavorable electrostatics (+21.6 kcal/mol) partially compensated by solvation (−17.6 kcal/mol), likely reflecting suboptimal orientation or disrupted salt–bridge interactions in the binding pocket.

**Table 1:** MM/GBSA Decomposition of Binding Free Energies for Ligands in MD Simulations of LCD with l-Lysine, l-Lysine Ethyl Ester, and Their Intermediate Complexes^a^

sample	ligand free energy (MM/GBSA)	electrostatic interaction	hydrogen bonding	hydrophobic interaction	van der Waals interaction	solvation interaction
LCD–l-lysine	–33.0 ± 3.4	–23.2 ± 3.0	–3.3 ± 0.2	–5.6 ± 1.3	24.5 ± 1.7	22.7 ± 2.7
LCD–l-lysine intermediate	–31.9 ± 2.8	21.6 ± 0.9	–2.4 ± 0.2	–7.0 ± 1.0	27.7 ± 0.8	–17.6 ± 0.9
LCD–l-lysine ethyl ester	–38.3 ± 2.8	–140.2 ± 15.4	–3.0 ± 0.2	–8.4 ± 0.2	–27.9 ± 1.6	140.1 ± 16.5
LCD–l-lysine ethyl ester intermediate	–49.8 ± 1.5	–38.6 ± 0.8	–1.9 ± 0.4	–9.3 ± 0.5	–32.2 ± 0.9	30.4 ± 1.1

a All energy terms are presented as mean ± standard deviation over three replicate MD simulations (kcal/mol). Not all the energy terms from the MM/GBSA calculations are listed.

Overall, these results indicate that ligand stability is driven primarily by van der Waals and hydrophobic interactions with electrostatic contributions modulated by solvation. Hydrogen bonding provides additional support, while the balance between electrostatics and desolvation explains how ligands with strongly charged interactions are stabilized in the active site.

### 2.2 Engineering the LCD Substrate Tunnel

Previous studies have identified residues D236 and E264 as key contributors to substrate delivery and product release.^11^ Our protein–ligand contact histograms, which were obtained from MD simulations, confirmed strong interactions between these residues and both l-lysine and l-lysine ethyl ester (see Figure S1). To reduce steric hindrance and enhance favorable interactions between l-lysine ethyl ester and the LCD substrate delivery tunnel, we individually mutated D236 to C and E264 to T, two smaller uncharged amino acids.

After the mutations, Caver analysis pointed out changes in the primary substrate tunnel of LCD (shown in Figure 5a), highlighted by the black box. In LCD (I61V–I94V), the primary tunnel is lined by residues R49, V61, E63, A235, D236, L237, and E264. In LCD (I61V–I94V-D236C), the primary tunnel involves residues R49, V61, E63, D236, Q260, E264, and T302. In LCD (I61V–I94V-E264T), the primary tunnel involves residues R49, V61, E63, A235, D236, L237, Q260, E264, and T302. Quantitatively, the tunnel throughput increased from 0.578 for LCD (I61V–I94V) to 0.600 for LCD (I61V–I94V-D236C) and 0.644 for LCD (I61V–I94V-E264T). Conversely, the cost value decreased from 0.548 for LCD (I61V–I94V) to 0.511 for LCD (I61V–I94V-D236C) and 0.440 for LCD (I61V–I94V-E264T). Here, throughput is a pathway importance score, ranging from 0 to 1, where lower-cost pathways have higher throughput and are therefore considered more important. Pathway cost reflects path quality, where lower cost corresponds to a more favorable pathway.^14^ Therefore, these results indicate that the tunnels in the LCD mutants represent more energetically and structurally preferred pathways compared to the wild type, characterized by smoother and less restrictive tunnel profiles, potentially arising from reduced steric clashes or improved residue packing along the tunnel lining. The top view of the LCD substrate tunnels and the locations of the mutated residues are shown in Figure 5b,c.

**Figure 5:**
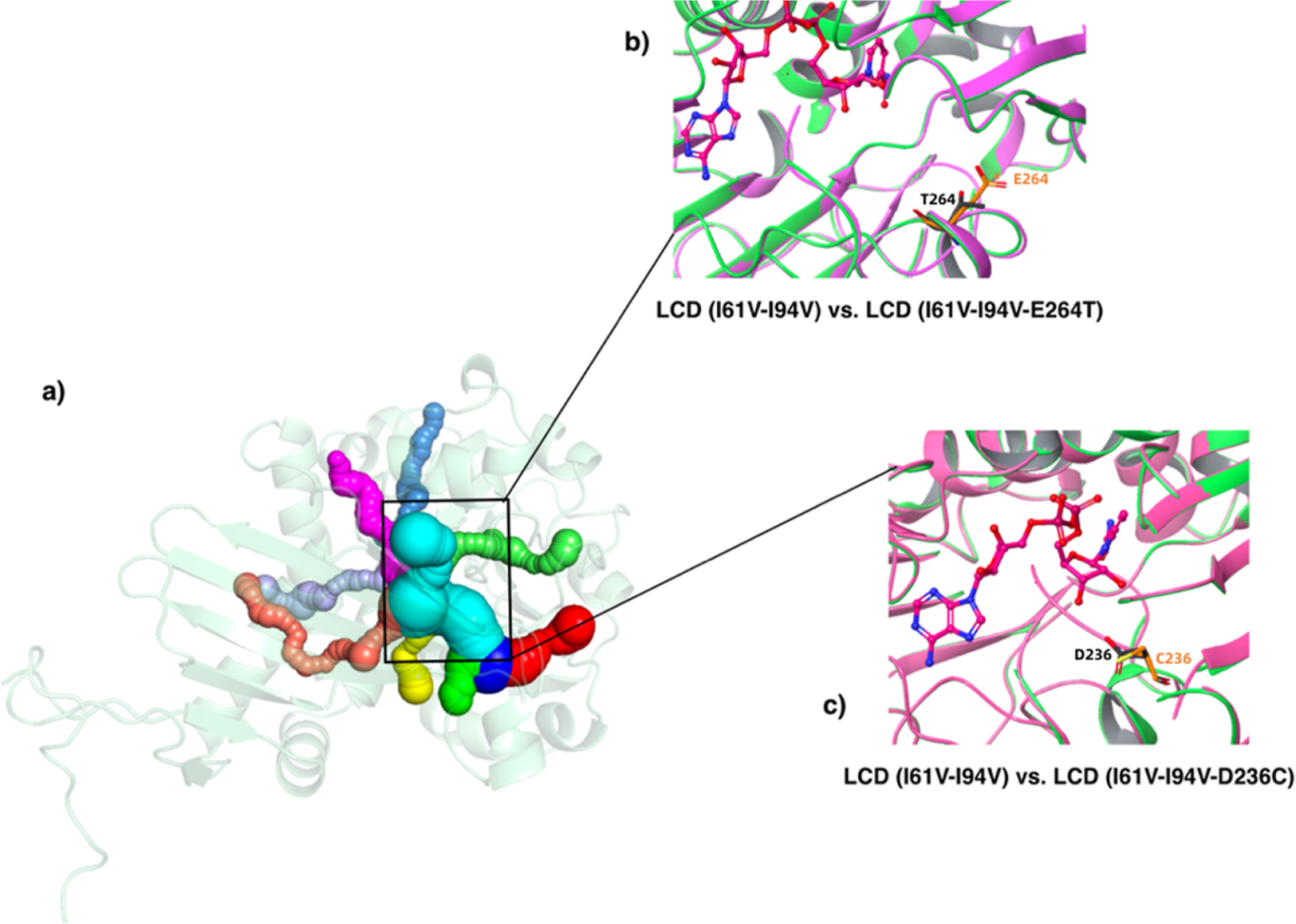
(a) Ten Caver-identified substrate tunnels of LCD (I61V–I94V), different colors are used to distinguish the tunnels identified by CAVER. The primary substrate tunnel is highlighted with a black box. (b) Top view of residue 26 positions in LCD (I61V–I94V) vs LCD (I61V–I94V-E264T). (c) Top view of residue 236 for LCD (I61V–I94V) vs LCD (I61V–I94V-D236C). NAD + is shown in magenta.

### 2.3 Mutagenesis, Expression, and Purification of SpLCD Variants

The proposed mutations were introduced by site-directed mutagenesis in the gene encoding the double mutant *Sp*LCD (I61V−I94V) available in our laboratory.^12^ The plasmid included an N-terminal His-tag to allow purification by affinity chromatography. Positions 236 and 264 were selectively mutated into the template, resulting in two distinct triple mutants, *Sp*LCD (I61V−I94V-D236C) and *Sp*LCD (I61V−I94V-E264T)

The resulting plasmids were transformed into *Escherichia coli* BL21­(DE3) Star cells, and protein expression and purification were performed using the conditions reported by Stalder et al.^12^ (Figure 6).

**Figure 6:**
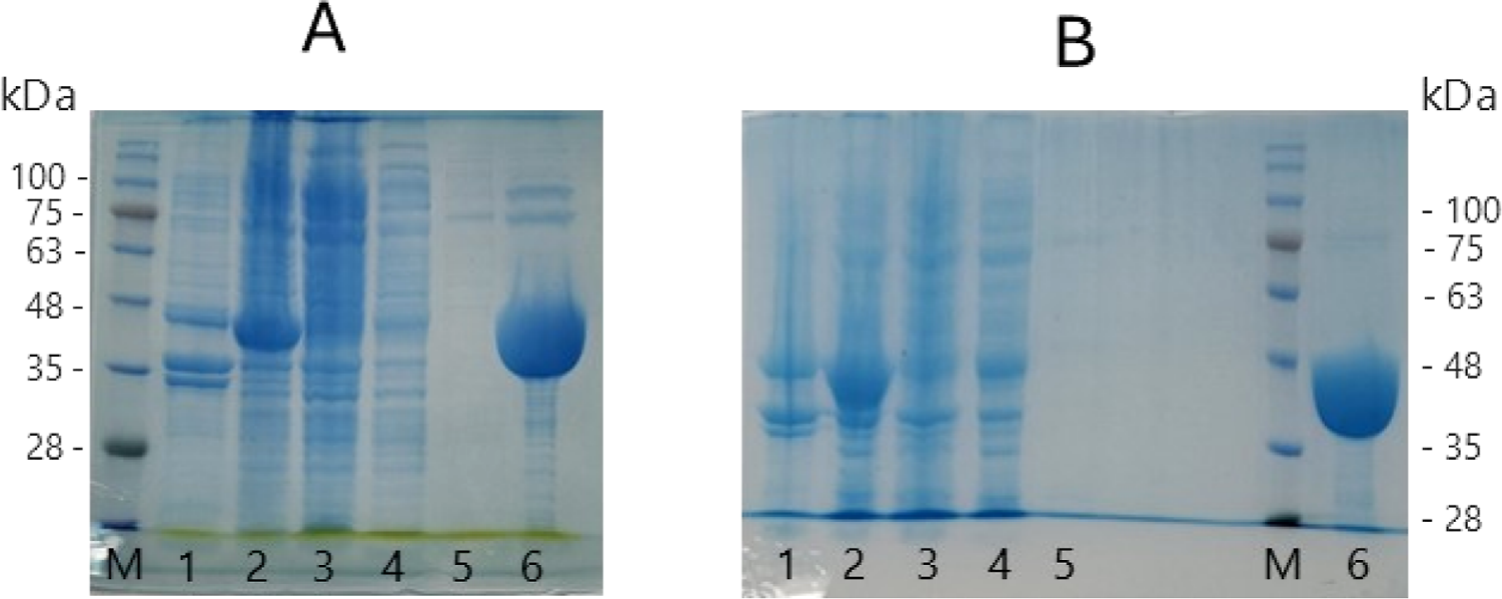
SDS-PAGE gels showing the purification stages of (A) SpLCD I61V–I94V-E264T and (B) SpLCD I61V–I94V-D236C. The gels illustrate protein purity throughout the purification process, confirming successful isolation of the target variants. M: marker; (1) pellet; (2) crude extract; (3) flowthrough; (4) first wash with 10 mM imidazole; (5) second wash with 10 mM imidazole; (6) purified protein eluted with 300 mM imidazole. Loading buffer: 50 mM phosphate buffer, 0.1 M NaCl, 10 mM imidazole, pH 7. Elution buffer: 50 mM phosphate buffer, 0.1 M NaCl, 300 mM imidazole, pH 7. Storage buffer: 50 mM phosphate buffer, 10% glycerol, pH 7.

The biomass yield for both triple mutants was approximately 6 g/L, a comparable value to that reported for *Sp*LCD (I61V−I94V) (7 g/L). However, the volumetric yields for *Sp*LCD (I61V−I94V-D236C) and *Sp*LCD (I61V−I94V-E264T) were 120 and 85 mg/L, respectively, both considerably lower than the yield reported for the double mutant I61V–I94V (250 mg/L) (Table 2). Despite the reduction in volumetric yield, the overall soluble expression and purification profiles of both variants were satisfactory, as shown in Figure 6.

**Table 2:** Expression Parameters for the SpLCD Variants Tested

SpLCD variant	biomass yield (g/L)	volumetric yield (mg/L)	protein concentration after dialysis (mg/mL)
I61V–I94V-D236C	6.1	120	8.5
I61V–I94V-E264T	5.7	85	7.5

### 2.4 Biotransformation Assays of *Sp*LCD Variants

The purified *Sp*LCD variants were tested in small-scale batch biotransformations to assess their catalytic activity with l-lysine and l-lysine ethyl ester as substrates. The ethyl ester substrate was chosen to be tested experimentally as it was employed in the computational design of the variants. The ester derivative stock solution was freshly prepared to minimize hydrolysis prior to the reaction. Reaction conditions were adapted from those described by Stalder et al.^12^ Biotransformations were performed at 10 mM substrate concentration in a final reaction volume of 0.5 mL and incubated at 37 °C with shaking. The enzyme concentration in each biotransformation was 1 mg/mL with l-lysine as a substrate or 3 mg/mL with its ethyl ester derivative to account for a potentially lower catalytic efficiency with the non-natural substrate. Samples were collected at different time points and analyzed by RP-HPLC or LC–MS after FMOC derivatization to allow compound detection at UV.

To assess the potential impact of spontaneous hydrolysis on the interpretation of biotransformation results, the hydrolysis rate of the ethyl ester substrate was monitored over time under the same reaction conditions as those used for the enzymatic transformation. This substrate-only negative control was essential to establish a reference for the expected nonenzymatic conversion of the ester to l-lysine (Figure 7). This comparison is particularly relevant, as the observed decrease in ester concentration during enzymatic assays could result from both enzyme-catalyzed conversion and spontaneous hydrolysis in the aqueous environment.

**Figure 7:**
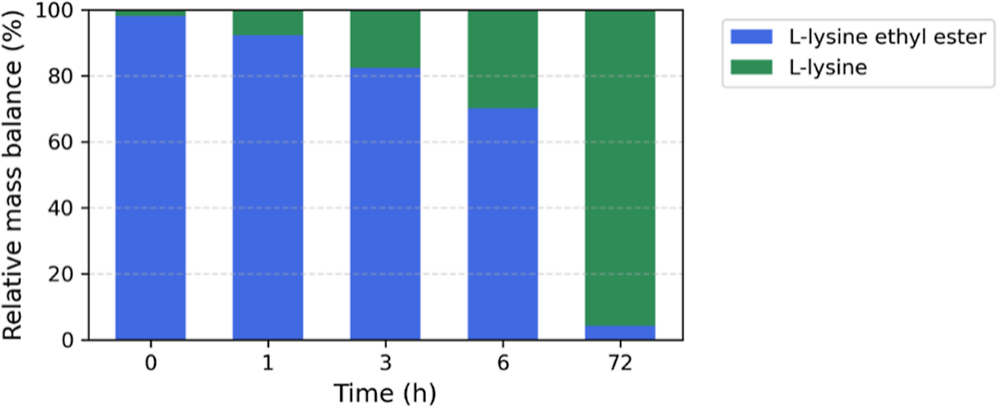
Relative mass balance of l-lysine ethyl ester and l-lysine over time under reaction conditions in the absence of an enzyme. The reaction was performed in 100 mM phosphate buffer (pH 7.0) at 37 °C with shaking (150 rpm). The graph shows the progressive spontaneous hydrolysis of the ester substrate in aqueous buffer under reaction conditions. Concentrations were determined by HPLC after FMOC derivatization and normalized to the total signal at each time point.

Another reference, for comparison, of the triple mutants consisted in testing in parallel the original double mutant *Sp*LCD (I61V−I94V) with l-lysine ethyl ester under the same reaction conditions. This variant, which has been already extensively characterized in our group, was included here to probe its ability to convert the ester substrate. Preliminary testing^15^ suggested that *Sp*LCD (I61V–I94V) was exclusively active on l-lysine produced by spontaneous hydrolysis of the ester. However, no analytical confirmation was provided.

In this work, this hypothesis was systematically confirmed by using HPLC and LC–MS techniques. As shown in Figure 8, the enzyme efficiently catalyzed the conversion of l-lysine to l-pipecolic acid once l-lysine ethyl ester was hydrolyzed. No traces of L-pipecolic ethyl ester were detected by HPLC at any time point.

**Figure 8:**
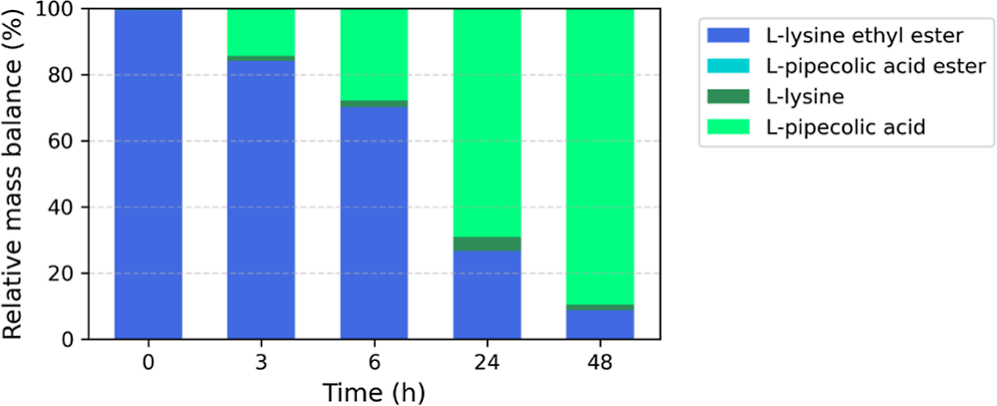
Relative mass balance at different time points for the reaction with SpLCD I61V–I94V and l-lysine ethyl ester as a substrate. Reaction conditions were: 3 mg/mL of enzyme, 10 mM of substrate in 100 mM phosphate buffer (pH 7), 37 °C and 150 rpm. Concentrations were determined by HPLC after FMOC derivatization and normalized to the total signal at each time point.

To further confirm these findings, LC–MS analysis was performed. Table 3 summarizes the expected and observed masses of relevant compounds in the reaction mixture at 6 h. The absence of detectable masses corresponding to l-lysine and L-pipecolic ethyl ester further supports the conclusion that the ester is initially hydrolyzed to l-lysine, which is then rapidly converted to l-pipecolic acid by the enzyme.

**Table 3:** LC–MS Confirmation of the Absence of l-Lysine and l-Pipecolic Ethyl Ester in the Reaction Mixture with SpLCD I61V–I94V after 6 h^a^

compound	expected mass	observed mass
l-Lysine ethyl ester	619.3 (M + H^+^)	619.3
	641.3 (M + Na^+^)	641.2
l-Lysine	591.3 (M + H^+^)	n.d.
	613.3 (M + Na^+^)	n.d.
l-Pipecolic acid ethyl ester	380.2 (M + H^+^)	n.d.
	402.2 (M + Na^+^)	n.d.
l-Pipecolic acid	352.2 (M + H^+^)	352.1
	374.2 (M + Na^+^)	374.1

a The expected and observed masses of relevant FMOC-derivatized adducts in the positive ion mode are reported.

The enzyme variant *Sp*LCD (I61V−I94V-D236C) showed no conversion of the natural substrate l-lysine to the corresponding product, l-pipecolic acid, even after 72h of incubation (data not shown). This suggests that the introduction of a mutation at position 236 may have led to complete inactivation of the enzyme toward its natural substrate.

Despite the lack of activity with l-lysine, this variant was also tested with l-lysine ethyl ester as a substrate under the same reaction conditions. The reaction profile, shown in Figure 9, closely mirrors that of the substrate-only negative control, indicating that no enzymatic conversion to L-pipecolic ethyl ester or l-pipecolic acid was observed. The only observable change over time was the progressive hydrolysis of the ester to l-lysine, with nearly complete depletion after 72 h. This result further supports the hypothesis that mutating the aspartic acid at position 236 to a cysteine significantly impairs the catalytic function of *Sp*LCD.

**Figure 9:**
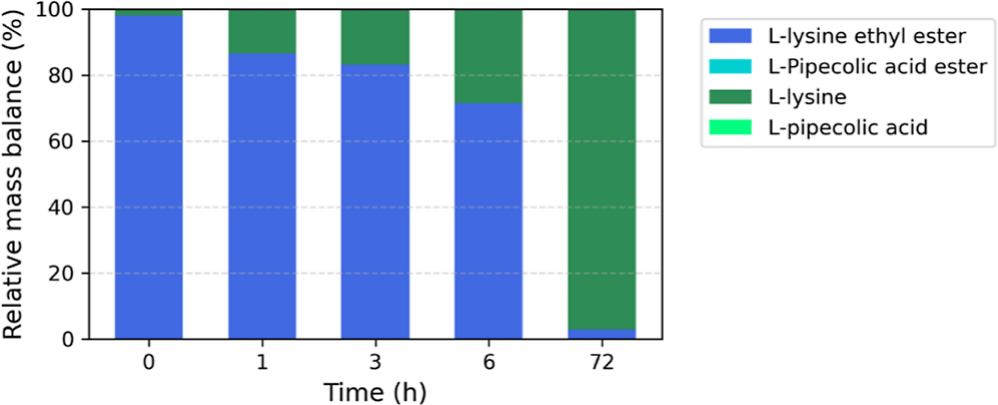
Relative mass balance at different time points for the reaction with SpLCD I61V–I94V-D236C with l-lysine ethyl ester as a substrate. Reaction conditions were: 3 mg/mL of enzyme, 10 mM of substrate in 100 mM phosphate buffer (pH 7), 37 °C and 150 rpm. No products, L-pipecolic ethyl ester or l-pipecolic acid, could be detected through HPLC analysis.

The triple mutant SpLCD (I61V−I94V-E264T) was also tested in the same conditions. The resulting mass balances, derived from HPLC analysis after FMOC derivatization, are displayed in Figures 10 and 11.

**Figure 10:**
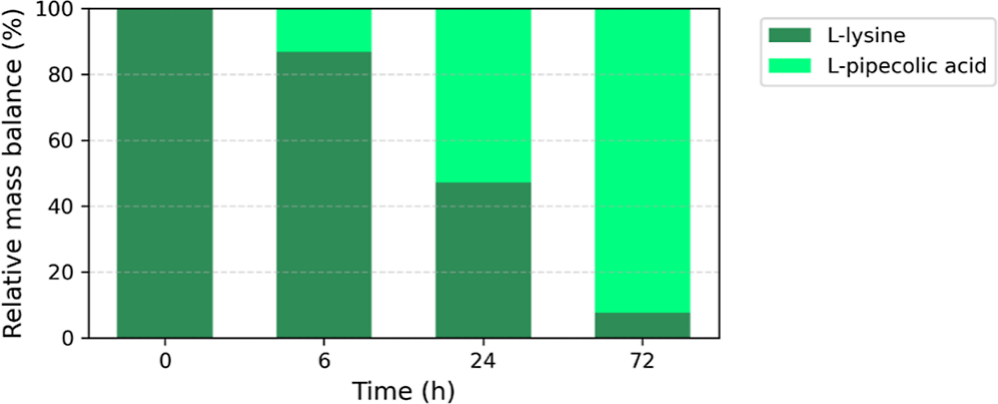
Relative mass balance at different time points for the reaction with SpLCD I61V–I94V-E264T with l-lysine as a substrate. Reaction conditions were: 1 mg/mL of enzyme, 10 mM of substrate in 100 mM phosphate buffer (pH 7), 37 °C and 150 rpm. Concentrations were determined by HPLC after FMOC derivatization and normalized to the total signal at each time point.

**Figure 11:**
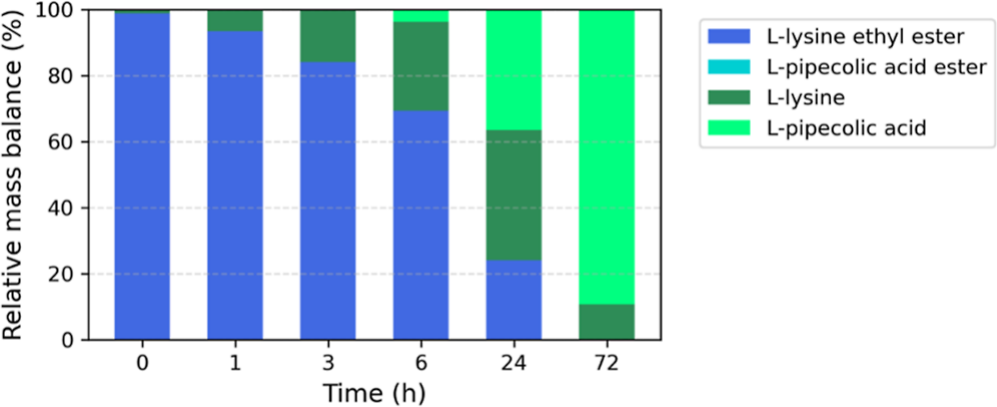
Relative mass balance at different time points for the reaction with SpLCD I61V–I94V-E264T with l-lysine ethyl ester as a substrate. Reaction conditions were: 3 mg/mL of enzyme, 10 mM of substrate in 100 mM phosphate buffer (pH 7), 37 °C and 150 rpm. Concentrations were determined by HPLC after FMOC derivatization and normalized to the total signal at each time point.

When l-lysine was used as a substrate, the enzyme could convert it to the corresponding product, reaching approximately 90% conversion after 72 h. At 6 h, however, the conversion was less than 20%, indicating a significantly lower catalytic efficiency compared to the original double mutant. Similar setup with 1 mg/mL of SpLCD I61V–I94V resulted in approximately 50% conversion at 6 h, with complete conversion achieved in 24 h.^15^ This comparison suggests that the addition of a third mutation in position 264 likely reduced the efficiency toward the natural substrate.

Again, when l-lysine ethyl ester was used as a substrate, no formation of L-pipecolic ethyl ester was detected throughout the 72 h reaction period, as shown Figure 11. Instead, at 72 h, the relative mass balance was almost entirely given by l-pipecolic acid, with only 10% of l-lysine remaining from ester hydrolysis. Similar to the double mutant, the variant I61V–I94V-E264T only converted l-lysine to l-pipecolic acid after ester hydrolysis, with no direct conversion of the ester to the corresponding product. The lower catalytic efficiency of SpLCD (I61V−I94V-E264T) compared to the double mutant is further highlighted by the higher residual concentrations of l-lysine observed at different time points (Figures 11 and 8).

The absence of l-pipecolic ethyl ester as a product was further confirmed by LC–MS analysis. Samples taken at 6 and 24 h were FMOC derivatized and analyzed to detect the potential esterified product. Table 4 compares the observed masses with the expected masses of FMOC-derivatized adducts in the reaction mixture. The presence of l-lysine adducts at both time points can be viewed as a further confirmation of the reduced catalytic efficiency of SpLCD I61V–I94V-E264T in producing l-pipecolic acid compared to the double mutant.

**Table 4:** LC–MS of the Reaction Mixture with SpLCD I61V–I94V-E264T (6 h)^a^

compound	exact mass	observed mass (6 h/24 h)
l-Lysine ethyl ester	619.3 (M + H^+^)	619.2/619.2
	641.3 (M + Na^+^)	641.3/641.2
l-Lysine	591.3 (M + H^+^)	591.2/591.2
	613.3 (M + Na^+^)	613.2/613.2
l-Pipecolic acid ethyl ester	380.2 (M + H^+^)	n.d./n.d.
	402.2 (M + Na^+^)	n.d./n.d.
l-Pipecolic acid	352.2 (M + H^+^)	352.1/352.1
	374.2 (M + Na^+^)	374.0/374.1

a The expected and observed masses of relevant FMOC-derivatized adducts in the positive ion mode are reported.

### 2.5 Exploring the LCD Catalytic Mechanism via Well-Tempered Metadynamics Simulations

Another hypothesis of why LCD cannot catalyze l-lysine ethyl ester to the final product is that the ethyl ester group prevents the oxidative deamination by direct cyclization, which is essential for the formation of pipecolic acid ester. To investigate whether the reaction is possible for both l-lysine and l-lysine ethyl ester, we analyzed the conformational dynamics of four ligand systems: l-lysine, l-lysine iminium intermediate, l-lysine ethyl ester, and l-lysine ethyl ester iminium intermediate (Figure 3). The inclusion of l-lysine and l-lysine ester aims to provide a negative control to verify that, without the oxidation to their respective iminium intermediates, the geometric conformations favorable for cyclization are not explored, as expected. Well-tempered metadynamics were employed to explore their conformational changes using two collective variables (CVs), which are shown in Figure 12: CV1, the distance between the nucleophilic nitrogen and the attacked Cα atom, and CV2, one of the dihedral angles associated with ring-closure geometry.

**Figure 12:**
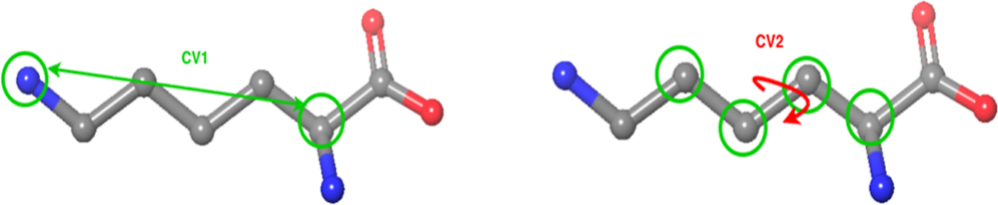
The two defined collective variables for well-tempered metadynamics. CV1 (Left): the distance between the nucleophilic nitrogen and the attacked Cα atom. CV2 (Right): the dihedral angle considered to describe the cyclization ring closure in the reaction.

To evaluate the propensity to cyclization in the four considered systems: l-lysine and l-lysine ethyl ester and their iminium intermediates, we monitored the distance between the Nε atom and the attacked Cα atom across all MD frames in which the ligand remained bound to the enzyme’s active site. Short Nε–Cα distances (≤3.5 Å) were used as indicators of a geometry compatible with nucleophilic attack. For each simulation, we report the number of frames in which the Nε–Cα distance falls within 3.0 Å, 3.5 Å, and 4.0 Å, as well as the corresponding number of frames adopting a product-like dihedral angle based on the LCD–l-pipecolic acid experimentally crystallized structure (PDB: 5GZM) (Figure 13, 57° ± 15°). Each LCD–ligand system performed 5 replica simulations, and each simulation consisted of 3000 frames, and the results are summarized in Table 5.

**Figure 13:**
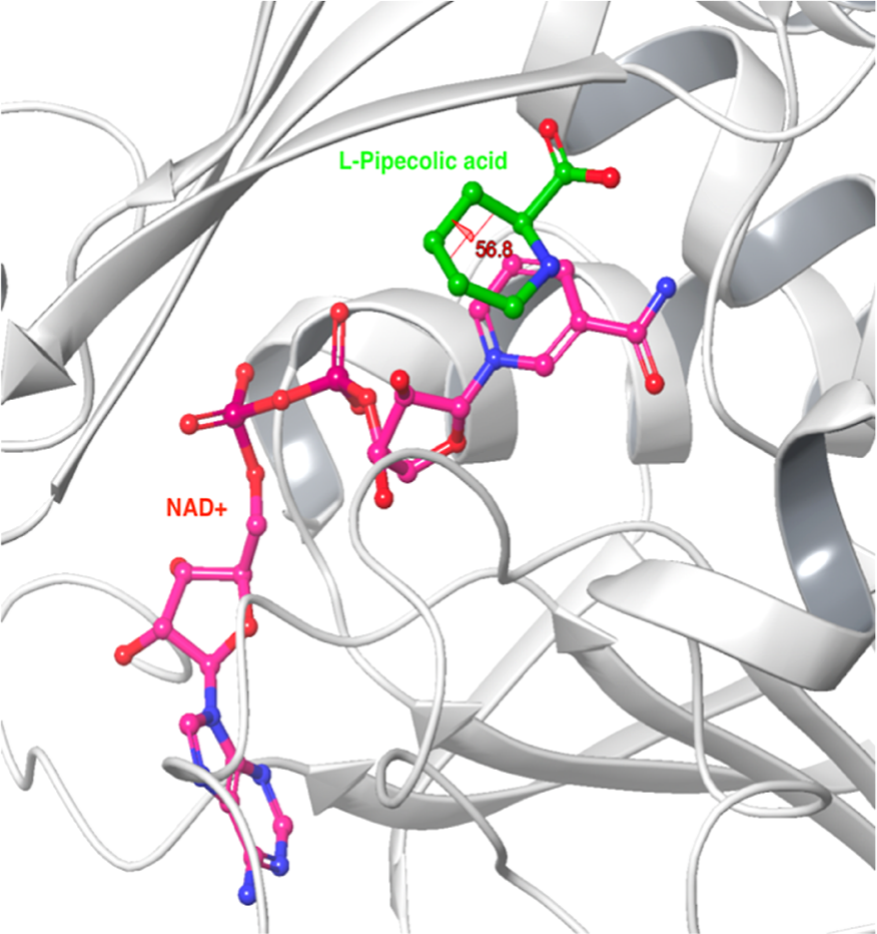
LCD–product (l-pipecolic acid) complex. The dihedral angle of l-pipecolic acid is highlighted.

**Table 5:** Nε–Cα Distances Sampled during MD Simulations for l-Lysine, l-Lysine Ethyl Ester, and Their Iminium Intermediate (Total Frames per Simulation = 3000)

	distance (≤3.0 Å)		distance (≤3.5 Å)		distance (≤4.0 Å)	
sample	num of frames	num of frames within dihedral angle 57° ± 15°	num of frames	num of frames within dihedral angle 57° ± 15°	num of frames	num of frames within dihedral angle 57° ± 15°
l-lysine—1	2	0	83	12	259	39
l-lysine—2	4	0	122	17	398	64
l-lysine—3	5	2	99	20	285	45
l-lysine—4	2	0	82	13	290	35
l-lysine—5	1	0	95	17	310	46
l-lysine iminium intermediate—1	68	10	398	62	823	114
l-lysine iminium intermediate —2	107	22	512	85	1008	145
l-lysine iminium intermediate —3	30	6	290	37	750	105
l-lysine iminium intermediate —4	42	4	284	48	705	101
l-lysine iminium intermediate —5	47	9	291	59	655	115
l-lysine ethyl ester —1	3	1	96	14	403	49
l-lysine ethyl ester—2	5	0	88	17	323	45
l-lysine ethyl ester—3	0	0	8	0	62	5
l-lysine ethyl ester—4	0	0	20	5	181	39
l-lysine ethyl ester—5	0	0	28	9	169	43
l-lysine ethyl ester iminium intermediate −1	43	4	281	38	624	86
l-lysine ethyl ester iminium intermediate—2	64	11	374	51	768	105
l-lysine ethyl ester iminium intermediate—3	18	5	149	23	421	61
l-lysine ethyl ester iminium intermediate—4	13	0	131	9	402	32
l-lysine ethyl ester iminium intermediate—5	52	6	298	46	654	79

As expected, sampling of short Nε–Cα distances by l-lysine is limited. Across five independent trajectories, only 1–5 frames exhibit Nε–Cα distances ≤3.0 Å. Even under relaxed geometric criteria, sampling remains sparse, with only 82–122 frames ≤3.5 Å and 259–398 frames at ≤4.0 Å. Only a small subset of these conformations simultaneously satisfies the ligand backbone carbon–carbon dihedral angle requirement (57° ± 15°), indicating that the native substrate rarely accesses the cyclization geometries. A similar behavior has been observed for l-lysine ethyl ester itself, exhibiting extremely limited sampling of Nε–Cα distances. At most, 0–5 frames satisfy the ≤3.0 Å criterion across simulations, with essentially no frames simultaneously meeting the dihedral requirement. Although the number of frames increases modestly at ≤3.5 Å (8–96 frames) and ≤4.0 Å (62–403 frames), conformations with product-like dihedral angles remain rare (0–17 frames ≤3.5 Å, 5–49 frames at ≤4.0 Å).

Differently, the l-lysine iminium intermediate shows a marked increase in both the frequency and quality of reactive conformations. Each trajectory samples 30–107 frames with Nε–Cα distances ≤3.0 Å, 284–512 frames at ≤ 3.5 Å, and 655 – 1008 frames at ≤4.0 Å. A substantial subset of these frames also satisfies the dihedral angle criterion (4–22, 37–85, and 101–145 frames, respectively), suggesting that iminium formation promotes the concerted attainment of short Nε–Cα distances and product-like torsional alignment. For the l-lysine ethyl ester iminium intermediate, conformations with Nε–Cα distances ≤3.0 Å are sampled 13–64 frames, while only 0–11 frames also meet the dihedral angle criterion. At broader cutoffs, 149–374 frames at ≤3.5 Å and 402–768 frames at ≤4.0 Å are observed, but the fraction of product-like dihedral angles remains modest low (9–51 and 32–105 frames, respectively).

These results suggest that esterification disfavors the formation and stabilization of cyclization-competent conformations for the proposed iminium intermediate. As expected, l-lysine and l-lysine ethyl ester, used as controls, are much farther away with respect to the cyclization geometry than their corresponding iminium intermediates.

Consistent with above observations, the free-energy plots for CV1 (ligand Nε–Cα distances ≤3.5 Å and ≤4.0 Å), derived from well-tempered metadynamics simulations, are shown in Figure 14. All the free-energy landscapes of the entire well-tempered metadynamics simulations are shown in Figure S3. These free-energy plots indicate that the LCD–l-lysine iminium system exhibits a generally lower energy profile, particularly when CV2 (the ligand backbone carbon–carbon dihedral angle within 57° ± 15°) is simultaneously satisfied during the simulations.

**Figure 14:**
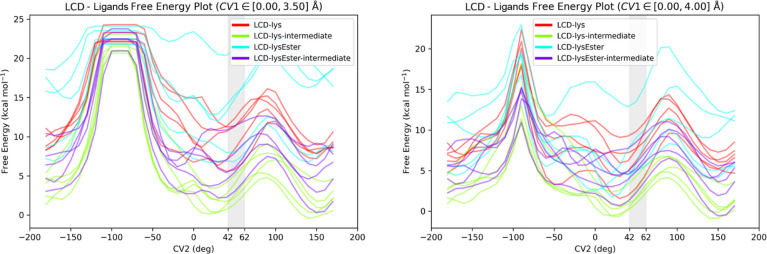
Free-energy surface (FES) obtained from LCD metadynamics simulations. Left: CV1 ≤3.5 Å; Right: CV1 ≤4.0 Å. The range of CV2 within 57° ± 15° is highlighted.

Taken together, we evaluated the reaction from three complementary perspectives: (i) frequent sampling of short Nε–Cα distances, (ii) alignment of the relevant dihedral angles toward a product-like geometry, and (iii) enhanced energetic stabilization of these reactive conformations. Under the proposed catalytic scenario, where both substrates are first converted into their respective iminium intermediates, the l-lysine iminium intermediate shows the most favorable behavior, as it most consistently satisfies all three criteria simultaneously during the simulations.

## 3 Materials and Methods

### 3.1 Protein and Ligand Preparation

The experimentally solved 3D structure of LCD (PDB ID: 5GZM) was obtained from the Protein Data Bank (https://www.rcsb.org/). Only chain A was retained for further in silico studies. The protein structure was prepared using the Protein Preparation Wizard program in the Schrödinger Maestro Suite (Release 2024-2).^16^ Missing hydrogen atoms were added consistency with the real experimental pH value of 7.0, and crystallographic water molecules were removed. All atoms were restrained to a maximum displacement of 0.3 Å, followed by energy minimization using the OPLS4 force field.

The 3D conformations of l-lysine and l-lysine ethyl ester were obtained from the PubChem database (https://pubchem.ncbi.nlm.nih.gov/) and prepared using the LigPrep program in Maestro. Protonation states were generated at pH 7.0 using Epik Classic, and geometries were minimized with an OPLS4 force field. The top-ranking conformers were selected for subsequent in silico studies.

### 3.2 In Silico Enzyme Mutagenesis

The LCD variant (I61V–I94V) was generated using the Residue Scanning Calculations module in the Schrödinger Maestro Suite (Release 2024-2). Residues I61 and I94 of the wild-type LCD were simultaneously mutated to valine under the side-chain prediction with backbone minimization option (cutoff = 0 Å). The additional single mutations D236C and E264T wer generated based on the LCD variant (I61V–I94V). The resulting mutant structures were further refined using the same protein preparation protocol described above.

### 3.3 Enzyme–Ligand Complexes Construction

The Induced Fit Docking (IFD) program in the Schrödinger Maestro Suite (release 2024-2) was utilized to construct the LCD–l-lysine and LCD–l-lysine ethyl ester complexes. Docking grids were centered on the cocrystallized ligand (l-pipecolic acid) with a 20 Å box size. SMARTS pattern constraints were applied to the four backbone carbon atoms of the ligands. Residues within 5 Å of each docked ligand were refined using Prime, and final poses were redocked using Glide (SP precision). The top-scoring complexes were selected for subsequent MD simulations.

LCD–l-lysine iminium intermediate and LCD–l-lysine ethyl ester iminium intermediate complexes were generated by manually modifying the amine group protonation states of l-lysine and l-lysine ethyl ester, respectively. In the intermediate systems, the LCD cofactor NAD^+^ was also manually modified to NADH to reflect the corresponding reaction states. These complexes were reprepared following the procedure in Section 3.1.

### 3.4 Substrate Tunnel Analysis

LCD substrate tunnel analysis was conducted on PyMOL^17^ and identified with CAVER 3.03 plugin software.^14^ The cofactor NAD was set as the starting point for tunnel calculations. Detection parameters included a probe radius of 0.9 Å, shell radius of 3.0 Å, shell depth of 4.0 Å, and a clustering threshold of 3.5 Å.

### 3.5 MD Simulation System Building

All the protein–ligand complexes were solvated using the System Builder module in the Schrödinger Maestro Suite (release 2025).^18^ Each system was enclosed in a 15 Å SPC water box and neutralized with Na^+^ ions. The OPLS4 force field was applied to all the systems.

### 3.6 Classical MD Simulations

MD simulations of LCD with l-lysine, l-lysine ethyl ester, and their intermediates were performed for 100 ns in three replicas using the Desmond simulation engine in the Schrödinger Maestro Suite (release 2025) with the OPLS4 force field. Positional restraints were applied to the Cα atoms of residues V61, W62, E63, G80, Y81, S82, P83, S210, V211, A212, V213, and D236. All restraints were applied with a force constant of 5.0 kcal/mol and were imposed only during the production stage of the simulations. Ligand binding free energies (MMGB-SA) were obtained using the Schrödinger thermal_mmgbsa.py script (version 2025) on every 10th simulation frame. Protein and ligand RMSD and RMSF analyses were obtained using the Schrödinger trj_rmsd.py script (version 2025).

### 3.7 Metadynamics Simulations

Well-tempered metadynamics simulations for exploring the LCD catalytical mechanism, as described in Section 2.5, were performed in five replicas using the Desmond simulation engine in the Schrödinger Maestro Suite (release 2025) with the OPLS4 force field. Two CVs were defined for each ligand:

- CV1: distance between the nucleophilic nitrogen atom and the attacked Cα atom (Gaussian width = 0.05 Å).
- CV2: dihedral angle formed by the four carbon atoms involved in the reaction cyclization step (Gaussian width = 5.0°).

To prevent large-scale conformational changes of the enzyme active site and to ensure that the ligand remained consistently positioned within the active site for accurate monitoring the Nε–Cα distance. Several restraints were applied: (1) the distance between the C4 carbon atom of the NAD + ribose ring (or NADH in the intermediate systems) and the α-NH2 nitrogen atom of the ligand was restrained to within 5.0 Å; (2) the cofactor NAD^+^ (or NADH in the intermediate systems) was positionally restrained; and (3) positional restraints were applied to the Cα atoms of residues V61, W62, E63, G80, Y81, S82, P83, S210, V211, A212, V213, and D236. All restraints were applied with a force constant of 5.0 kcal/mol and were imposed only during the production stage of the simulations. Well-tempered metadynamics simulations were subsequently conducted in the *NPT* ensemble at 300 K. Gaussian height was set to 0.003 kcal/mol, and the deposition interval was set as 0.09 ps. The well-tempered metadynamics simulations were run for 400 ns per replica, with frames recorded every 100 ps (4000 frames per simulation), and the temperature factor (kTemp) was set to 5.

Postprocessing of simulation trajectories was performed for the first 300 ns only. During this period, the ligands’ Nε–Cα distances and the ligand backbone dihedral angles were calculated using the Measurement function in the Maestro graphical interface. Free-energy surface plots were produced mainly using pandas^19^ and matplotlib^20^ Python package.

### 3.8 Materials

All chemicals, reagents, and medium components were obtained as analytical grade from Sigma-Aldrich, Acros Organics, Apollo Scientific, and Fisher Scientific and were used without further purification. Primers were synthesized by Microsynth AG. All of the materials and kits used for mutagenesis were purchased from New England Biolabs (NEB). The *E. coli* strain BL21­(DE3) used in this work was purchased from Thermo Fisher Scientific.

### 3.9 Mutagenesis, Protein Expression, and Purification

Enzyme variants were generated by site-directed mutagenesis, using the pET-28b­(+) plasmid encoding the double mutant *Sp*LCD I61V–I94V gene as a template. Mutagenesis was performed using the Q5 Site-Directed Mutagenesis Kit (New England Biolabs), according to the manufacturer’s protocol (Table S2), for exponential amplification (Table S3), the KLD reaction, and subsequent transformation into competent cells.

Protein expression and purification were performed using the conditions reported by Stalder et al.^12^ Briefly, transformed cells were grown in LB medium supplemented with kanamycin (100 μg/mL) at 37 °C with shaking at 150 rpm. When the cultures reached an optical density at 600 nm (OD) of 0.6–0.8, protein expression was induced by adding 0.1 mM IPTG. The cultures were then incubated at 25 °C for 20 h. The cells were harvested by centrifugation at 2500 rpm for 20 min at 4 °C, and the enzymes were then purified manually by affinity chromatography using the High-Performance Ni Sepharose resin (Cytiva).

### 3.10 Batch Biotransformations

Biotransformations were performed in 100 mM phosphate buffer (pH 7), containing 10 mM substrate in a final reaction volume of 0.5 mL, incubated at 37 °C and 150 rpm. The enzyme concentration in each biotransformation was 1 mg/mL with l-lysine as a substrate or 3 mg/mL with its ethyl ester derivative to account for a potentially lower catalytic efficiency with the non-natural substrate. Samples were collected at different time points and analyzed by RP-HPLC or LC–MS. To allow the detection of compounds at UV, the samples were FMOC derivatized as reported by Stalder et al.^12^

### 3.11 HPLC Analysis

Samples were analyzed by HPLC using a Dionex UltiMate3000 (Thermo Fisher Scientific), equipped with a Waters X-BridgeC18 column (3.5 μm, 2.1 × 100 mm). Water containing 0.1% trifluoroacetic acid was used as eluent A, while MeCN containing 0.1% trifluoroacetic acid was used as eluent B. Chromatographic separation was performed at 0.8 mL/min with the following gradient: 0–1 min 95% A:5% B; 1–5 min 5% A:95% B; 5.1–6.6 min 100% B; 6.6–7 min 95% A:5% B; and 7–10 min 95% A:5% B. The FMOC-derivatized compounds were detected using UV detectors at 265 nm.

### 3.12 LC–MS Analysis

Samples were analyzed by LC–MS using an Agilent 1260 Infinity II LC system equipped with an Agilent SB-C18 column (1.8 μm, 2.1 mm × 50 mm) and an Agilent InfinityLab LC XT Mass Selective Detector (MSD). Ultrapure water containing formic acid (0.1%, v/v, solvent A) and ACN (solvent B) were employed as a mobile phase at a flow rate of 0.8 mL/min. A gradient of 0–7 min, 0 to 90% B, 7–10 min, 90% B, 10–11 min, 90 to 0% B, 11–14 min, 0% B was employed. The full scan was performed in both positive and negative ion modes in the range of *m*/*z* 100–1000.

## 4 Conclusion

This study investigates the catalytic mechanism and substrate recognition of l-lysine cyclodeaminase (LCD) through an integrated approach combining molecular docking, tunnel engineering, classical molecular dynamics simulations, well-tempered metadynamics, and experimental validation. Our results show that l-lysine, l-lysine ethyl ester, and their intermediates can be accommodated within and delivered to the LCD active site. Engineering of the LCD substrate tunnel yielded two variants, LCD (I61V−I94V−D236C) and LCD (I61V−I94V−E264T), which exhibited improved tunnel properties, including increased throughput and reduced cost values. However, experimental assays revealed that neither variant acquired the desired catalytic activity toward l-lysine ethyl ester. The LCD (I61V−I94V−D236C) variant showed complete loss of activity toward both the native substrate and its ethyl ester derivative, suggesting that residue D236 plays a critical role in substrate recognition or catalysis. In contrast, the LCD (I61V−I94V− E264T) variant retained partial activity toward l-lysine but was unable to catalyze the cyclodeamination of l-lysine ethyl ester. Despite this outcome, the study provides valuable insights into the functional roles of the targeted residues D236 and E264 in the catalytic activity of *S. pristinaespiralis* LCD. The absence of l-pipecolic acid ethyl ester formation was confirmed using optimized HPLC and LC–MS analyses. These results support the initial hypothesis that the ester substrate undergoes spontaneous hydrolysis to l-lysine prior to enzymatic conversion, yielding l-pipecolic acid without detectable formation of the esterified product.

Well-tempered metadynamics simulations further indicate that l-lysine and l-lysine ethyl ester fail to reach pseudocyclization geometries, as expected. The proposed l-lysine ethyl ester iminium intermediate exhibits less favorable geometric and energetic properties for the cyclization step compared with the l-lysine iminium intermediate. These findings support the limited capacity of LCD to efficiently catalyze l-lysine ethyl ester and help explain the experimentally observed lack of activity. Importantly, they inform future efforts to engineer LCD for the conversion of larger esterified substrates. One promising strategy involves designing LCD variants capable of retaining such substrates in the active site with geometries and energetic profiles conducive to catalysis, for which the well-tempered metadynamics framework developed here represents a powerful evaluation tool.

Overall, this work establishes a detailed mechanistic framework linking enzyme dynamics, substrate recognition, and catalytic efficiency of LCD. This framework provides valuable guidance for the rational engineering of LCDs and related enzymes to achieve broader substrate scopes and enhanced catalytic performance in industrial biotechnology. From a practical perspective, it should be noted that l-lysine ethyl ester is not a naturally occurring compound and is substantially more expensive than l-lysine (2.7 CHF/g versus 1.3 CHF/g). Consequently, its application in chemoenzymatic processes would be justified only if it conferred a clear synthetic advantage. At present, the enzymatic conversion of l-lysine to l-pipecolic acid, followed by chemical esterification, remains the most practical and cost-effective strategy.

## Supplementary Material



## Data Availability

Materials are archived in the University of Milan Dataverse (institutional FAIR repository) if applicable at the following link: 10.13130/RD_UNIMI/TNYZJN.

## Notes

Docking scores of LCD–l-lysine and LCD–l-lysine ethyl ester; PCR protocol used to perform site-directed mutagenesis; primers used to perform site-directed mutagenesis; protein–ligand contacts of LCD–l-lysine and LCD–l-lysine ethyl ester during simulations; mean profiles (protein Cα RMSD, ligand RMSD, protein residue-wise RMSF) from MD simulations for all studied systems, including LCD–l-lysine, LCD–l-lysine ethyl ester, and their intermediate complexes; and free-energy landscape obtained from LCD well-tempered metadynamics simulations (PDF)
