# Supplementary material for: Mechanistic Insights into Lysine Cyclodeaminase Catalysis
Source: ACS Omega. 2026 May 22;11(22):32751–61. doi: 10.1021/acsomega.6c01676 (PMC13261480; doi:10.1021/acsomega.6c01676)
Supplement: Supplementary file 1 [file ao6c01676_si_001.pdf]

Supplementary Information

Mechanistic Insights into Lysine Cyclodeaminase Catalysis

Yao Wei[1#], Beatrice Rassati[2#], Uliano Guerrini[1], Francesca Paradisi[2*], Ivano Eberini[1*]

[1]Dipartimento di Scienze Farmacologiche e Biomolecolari “Rodolfo Paoletti”, Università degli Studi di Milano, Via Giuseppe Balzaretti 9, 20133, Milano, Italy
[2]Department of Chemistry, Biochemistry and Pharmaceutical Sciences, University of Bern, Freiestrasse 3, 3012 Bern, Switzerland

[#] Yao Wei and Beatrice Rassati share equal contribution
* Corresponding authors: Ivano Eberini Ivano.eberini@unimi.it
Francesca Paradisi francesca.paradisi@unibe.ch

Table S1. Docking scores of LCD – L-lysine and LCD – L-lysine ethyl ester

Sample	Glide score	IFDScore
LCD – L-lysine	-5.993	-14160.62
LCD – L-lysine ethyl ester	-1.999	-14160.62

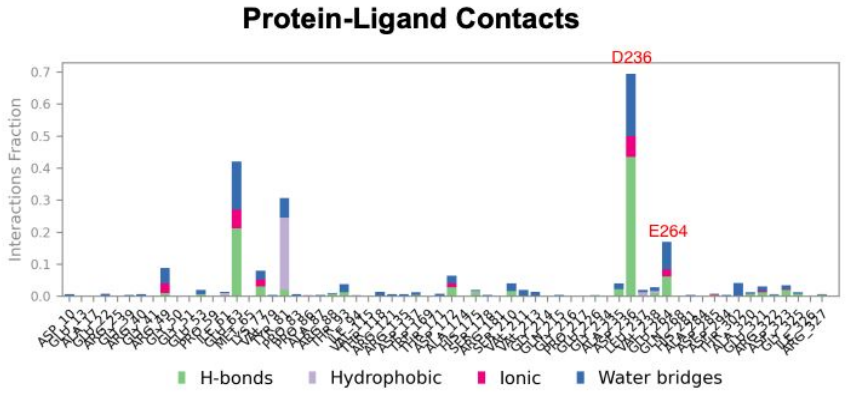

a)

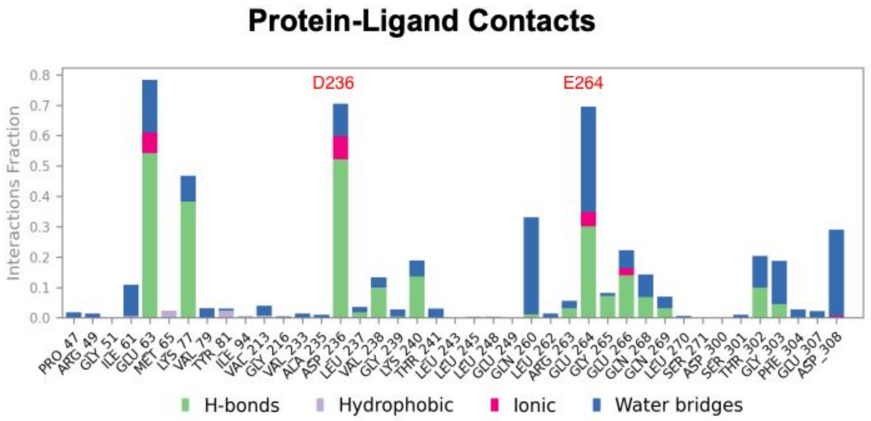

b)

Figure S1. Protein-ligand contacts during simulations: a) LCD – L-lysine, b) LCD – L-lysine ethyl ester

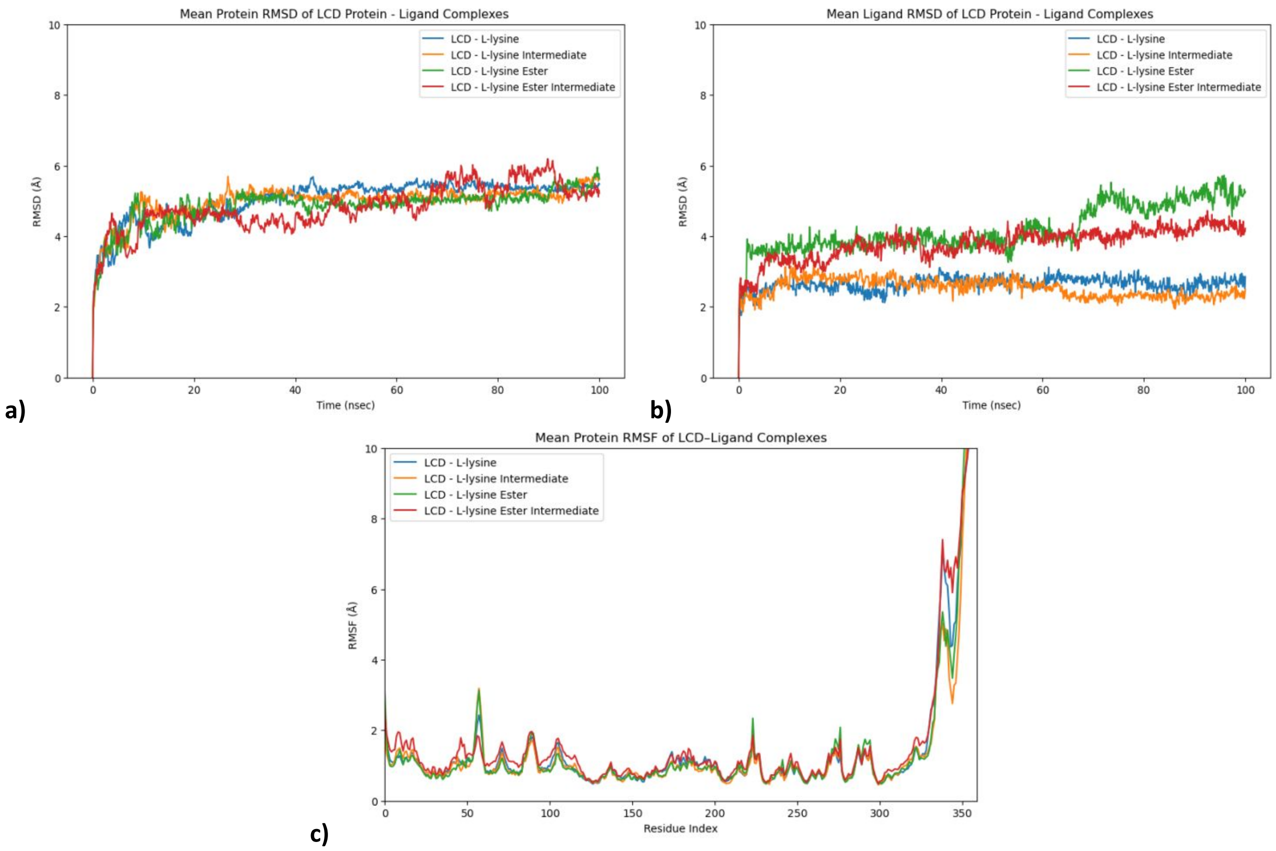

Figure S2. Mean profiles from MD simulations for all studied systems, including LCD – L-lysine, LCD – L-lysine ethyl ester, and their intermediate complexes: a) protein Cα RMSD; b) ligand RMSD; c) protein residue-wise RMSF. All curves represent the mean of three independent replicate simulations.

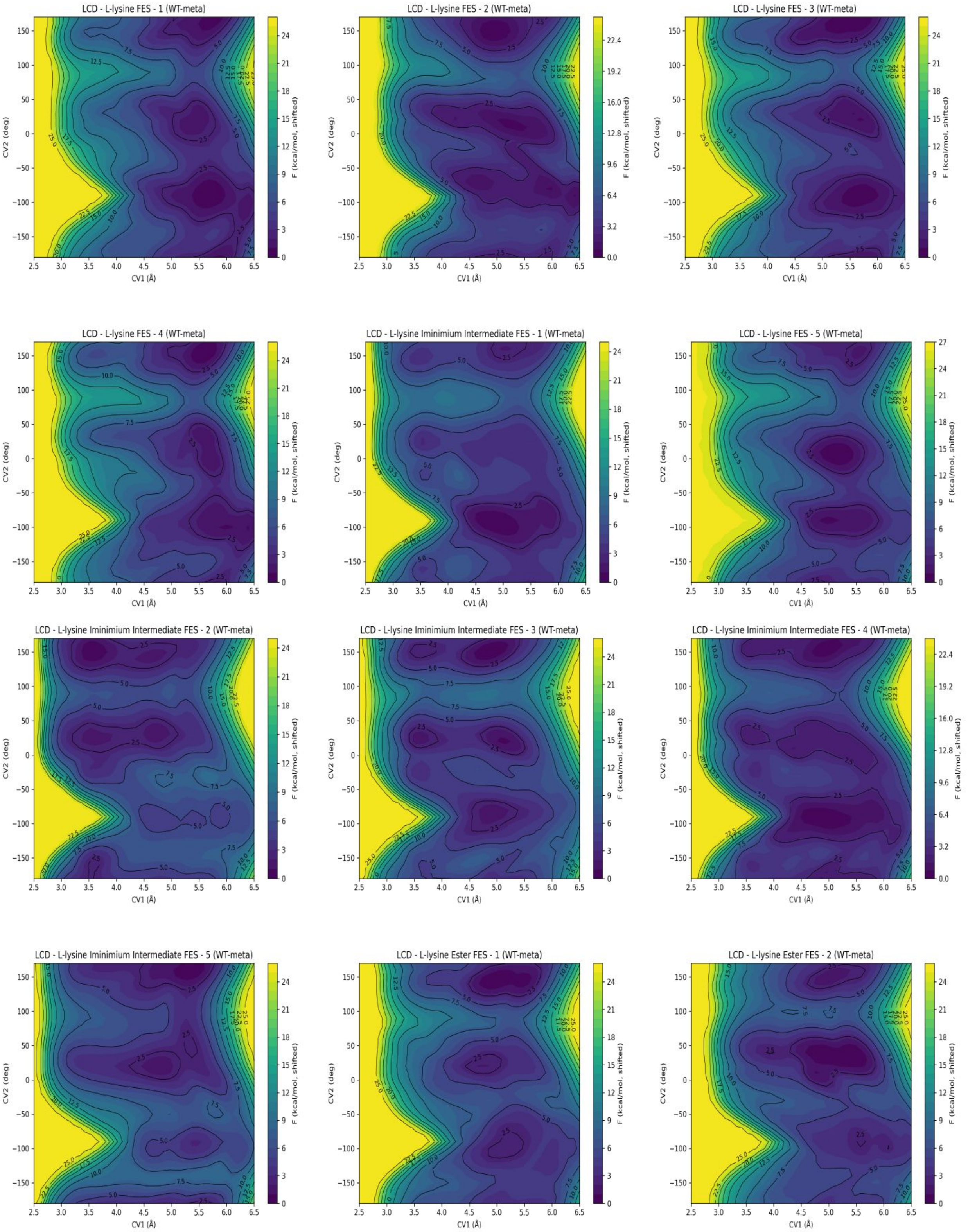
LCD - L-lysine FES - 1 (WT-meta)
LCD - L-lysine FES - 2 (WT-meta)
LCD - L-lysine FES - 3 (WT-meta)
LCD - L-lysine FES - 4 (WT-meta)
LCD - L-lysine Iminimium Intermediate FES - 1 (WT-meta)
LCD - L-lysine FES - 5 (WT-meta)
LCD - L-lysine Iminimium Intermediate FES - 2 (WT-meta)
LCD - L-lysine Iminimium Intermediate FES - 3 (WT-meta)
LCD - L-lysine Iminimium Intermediate FES - 4 (WT-meta)
LCD - L-lysine Iminimium Intermediate FES - 5 (WT-meta)
LCD - L-lysine Ester FES - 1 (WT-meta)
LCD - L-lysine Ester FES - 2 (WT-meta)
CV1 (Å)
CV2 (deg)
F (kcal/mol, shifted)

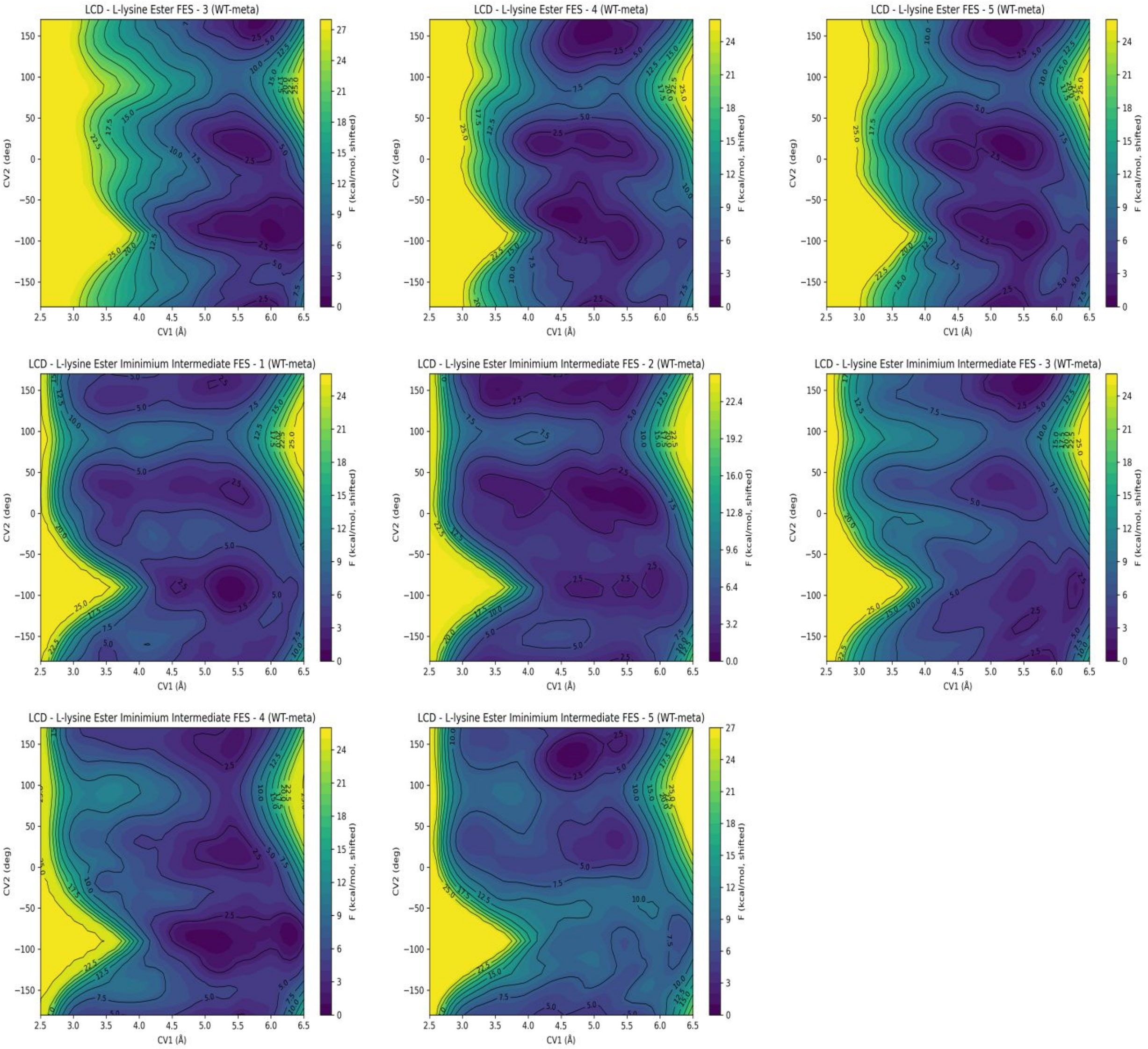

Figure S3. Free energy landscape obtained from LCD well-tempered metadynamics simulations

Table S2. PCR protocol used to perform site-directed mutagenesis.

Step		Temperature	Time
Initial denaturation		98°C	30 s
35 cycles	Denaturation	98°C	10 s
	Annealing	Tm of primers in **Table S4**	30 s

	Elongation	72°C	30 s/kb
Final extension		72°C	5 min

Table S3. Primers used to perform site-directed mutagenesis.

Primer	Sequence (5'-3')	Tm
*Sp*LCD D236C forward	GTGGGCGCATGTCTAGTGGGCAAAACC	63°C
*Sp*LCD D236C reverse	CGCATTGATATGCAGGTGCTCGCGAAC	65°C
*Sp*LCD E264T forward	GCCCTTCGGACGGGCGAATGTCAGCAA	66°C
*Sp*LCD E264T reverse	CTGCTCCGGATGGTCTGCGGTCACGAA	66°C
